# Electric vehicle adoption, gentrification, and housing prices: a longitudinal study in California

**DOI:** 10.1088/2515-7620/ae4b00

**Published:** 2026-03-10

**Authors:** Futu Chen, Sandrah P Eckel, Lawrence A Palinkas, Jill Johnston, Andre Comando, Alberto Campos, Wilma Franco, Erika Garcia

**Affiliations:** 1 Department of Population and Public Health Sciences, Keck School of Medicine, University of Southern California, Los Angeles, CA, United States of America; 2 Herbert Wertheim School of Public Health and Human Longevity Science, University of California, San Diego, CA, United States of America; 3 Department of Environmental and Occupational Health, Joe C. Wen School of Population and Public Health, University of California, Irvine, Irvine, CA, United States of America; 4 METRANS Transportation Consortium, Sol Price School of Public Policy, University of Southern California, Los Angeles, CA, United States of America; 5 Southeast Los Angeles Collaborative, Bell Gardens, CA, United States of America

**Keywords:** zero emission vehicles, electric vehicles, adoption, gentrification, housing, disparity

## Abstract

Increasing adoption of zero-emission vehicles (ZEVs) has raised concerns about potential disparities, including increased gentrification in low-income communities. As a first step in unraveling this rarely studied relationship, we examined the association between ZEV adoption and measures of gentrification. We addressed two questions: (1) How do ZEV adoption trends vary by neighborhood-level gentrification and disadvantage in California metropolitan areas? and (2) Among neighborhoods that are not already gentrified, is prior ZEV adoption associated with subsequent changes in housing prices? We obtained census tract-level data on longitudinal counts of battery electric vehicle (BEV) and plug-in hybrid electric vehicle (PHEV) registrations, median home value and monthly rent, disadvantaged communities (DACs) designations, and gentrification status (‘none/early,’ ‘advanced/stable,’ or ‘exclusive’). Linear mixed models estimated BEV (or PHEV) adoption trajectories (2015–2023) across gentrification status between DAC and non-DAC, allowing nonlinear time trends. Among ‘none/early’ gentrification tracts, we related prior ZEV adoption (2015–2019) to subsequent (2020–2023) home values and rental prices using linear mixed models. ZEV adoption trends varied by gentrification in non-DAC tracts, while DACs had lower levels of ZEV adoption and less evidence for disparities. Among ‘none/early’ gentrification tracts, tracts with higher BEV (or PHEV) adoption were associated with larger absolute increases in home value compared to tracts in the lowest adoption quartile. These early findings support community concerns that ZEV adoption may influence housing costs and gentrification. Further research is needed on these complex relations and possible policy interventions.

## 1. Introduction

The transition to zero-emission vehicles (ZEVs), including battery and plug-in hybrid electric vehicles (EVs), offers significant environmental and public health co-benefits [1–4]. Empirical studies have linked ZEV adoption with reduced exposure to tailpipe pollutants such as nitrogen dioxide (NO_2_) and lower asthma rates [1], particularly cases attributable to traffic-related air pollution (TRAP) [2].

Despite statewide growth in ZEV adoption, California’s transition has not been equitable, with adoption less likely to occur in disadvantaged neighborhoods [5]. Achieving equitable adoption, especially for historically marginalized communities, remains a challenge. Research found that areas with lower educational attainment— a proxy for socioeconomic status (SES) — experienced delayed ZEV uptake in California [1]. A study reported that ZEV ownership rates were nearly four times higher in non-disadvantaged communities (DAC) compared to DAC-designated areas in the Greater Los Angeles region [6]. Importantly, DACs face disproportionately high exposure to TRAP [7–9] and are more vulnerable to TRAP-related health impacts [10, 11]. Thus, the health of these communities is poised to benefit the most from the transition to electric transportation. Engaging disadvantaged communities is therefore critical for scaling up ZEV adoption and promoting environmental justice and health equity [3, 6].

The Electric Vehicle Adoption in California (EVAC) study is a community-based research project conducted in partnership with the Southeast Los Angeles Collaborative, a community-based organization serving local under-resourced communities. Annual meetings are held with a Community Advisory Council, comprising representatives from local and regional government agencies, community-based organizations, and advocacy groups [12]. A concern raised by these groups was the potential for investments relevant to ZEV infrastructure to accelerate gentrification and displacement, a process in which historically marginalized neighborhoods are transformed and repurposed by dominant socioeconomic or racial groups [13, 14]. In addition to investments that increase the local cost of living, the influx of higher-income populations—those who can afford high-priced housing and new vehicle technologies—into historically marginalized communities may further displace local residents [15, 16], making them more marginalized and adverse impact housing security.

Previous research has linked transportation investments and gentrification [17–20]; yet little work has incorporated real-world data on ZEVs. To date, only one California-based study [21] investigated the association between the installation of EV charging stations (EVCS) and increases in house sales price premiums (additional home value over comparable properties lacking access to EVCS). To address this gap, and to provide a first empirical characterization of this relationship, we used longitudinal census tract-level ZEV, median home value, and median monthly rental prices data from 2015 to 2023, as well as gentrification typologies, to answer the following questions: (1) Do disparities in ZEV adoption trends differ by urban gentrification typologies in California metropolitan areas?; and (2) Among neighborhoods that are non-gentrified or in the early stages of gentrification, are changes in ZEV adoption over the previous five years associated with subsequent changes in housing prices? The primary goal of this study is not causal identification, but to provide a first empirical characterization of how ZEV adoption patterns differ across neighborhoods with different gentrification trajectories, and how these patterns intersect with housing prices.

## 2. Methods

The study focused on two research questions to examine the relationship between gentrification, housing prices, and ZEV adoption. A flow chart (figure S.1) summarizes the two primary research questions and the corresponding analytical workflow. The sections below describe each data source and analytical step in detail.

### 2.1. Zero-emissions vehicles data

We obtained vehicle registration counts by census block group from the California Air Resources Board (CARB) Fleet Database [22], based on California Department of Motor Vehicles records. To match gentrification typologies’ geography, data were aggregated to the census tract level. Consistent with CARB and California Energy Commission’s definition [23], we use the term ZEV as an umbrella category. In this study, ZEVs include passenger light-duty battery electric vehicles (BEVs) and plug-in hybrid electric vehicles (PHEVs). Although California’s definition of ZEV includes fuel cell electric vehicles, we excluded them due to very low numbers. Importantly, BEVs and PHEVs rely on different propulsion technologies. BEVs are powered exclusively by electricity, while PHEVs combine an electric battery with an internal combustion engine. To reflect these differences, we conduct analyses separately by vehicle type. Annual counts of BEVs and PHEVs from 2015–2023 were summed and jointly referred to as ZEVs.

### 2.2. Disadvantaged communities (DAC)

We defined disadvantaged communities (DACs) following the California Environmental Protection Agency’s criteria under Senate Bill [24]. DACs were defined as communities in the highest 25^th^ percentile of CalEnviroScreen 4.0 [7], a screening tool released in May 2021 that integrates environmental pollution, health, and socioeconomic indicators into a cumulative burden percentile, with higher values indicating greater vulnerability [24].

### 2.3. Gentrification typology

Gentrification typologies were obtained from the Urban Displacement Project (UDP; [25] which describe trends in the housing market and potential for displacement or gentrification for census tracts in the San Francisco (SF) Bay Area and Southern California (Los Angeles (LA), Orange, and San Diego Counties) between 1990–2000 and 2000–2018, based on U.S. Census housing and demographic data, as well as Zillow home and rental values. Details are published elsewhere [26]. For this analysis, we grouped the nine original typologies (expanded in UDP documentation [25, 26]) into three broader gentrification statuses following prior literature [27]: (1) *None/Early* (‘Low-Income/Susceptible to Displacement,’ ‘Ongoing Displacement,’ ‘At Risk of Gentrification,’ ‘Early/Ongoing Gentrification’); (2) *Advanced/Stable* (‘Advanced Gentrification,’ ‘Stable Moderate/Mixed Income’); and (3) *Exclusive* (‘At Risk of Becoming Exclusive,’ ‘Becoming Exclusive,’ ‘Stable/Advanced Exclusive’). See table S.1 for details on the original typologies and their classification into these three statuses. Gentrification typology data were available for 6,447 California census tracts (80.0% of the state’s 8,057 census tracts; 80.4% of the total 39.15 million California population in 2018) covering the period 2000–2018 after we excluded census tracts with typologies of ‘High student population’ or ‘Unavailable or Unreliable Data’, N = 215 (3.2%). Figure S.2 illustrates the study area.

### 2.4. Housing prices and covariates

Census tract-level data on population characteristics and housing prices (2015–2023) were obtained from the American Community Survey (ACS) 5-Year Estimates [28], using the last year of each series as the reference. We included longitudinal median home value (US dollars, USD) and median monthly gross rent (USD). We selected housing prices as a proxy for the gentrification process because they are included in various gentrification measures [26, 29] and represent downstream consequences of gentrification [30]. Additional covariates included total population size, educational attainment (% aged ≥25 with at least a bachelor’s degree), and median household income [28].

### 2.5. Data processing

To describe ZEV adoption, we normalized counts by census tract population, described as the number of ZEVs per 1,000 population. To avoid instability of this value for tracts with very small populations, we excluded census tracts-years that had < 300 residents (n = 21 out of 58,023 census tracts-years with gentrification data, 0.04%). All data were aligned to 2010 census geography (consistent with UDP gentrification typologies) by crosswalking 2020 census data to 2010 boundaries using population weight [28]. For count variables, crosswalked values represent the sum of weighted 2020 census tract data; for medians, they represent the aggregated weighted sums.

For gentrification and DAC analysis, tracts missing DAC status (due to unavailable CalEnviroScreen data) were excluded, yielding 57,903 tract-year observations (2015–2023). For housing price analyses limited to None/early gentrification tracts, 7,893 tract-year observations (2020–2023) were available: 7,329 for home value models and 7,304 for rent models. Adjusting for median household income further reduced analytic samples to 7,304 and 7,787, respectively. Figure S.3 presents the flow chart.

### 2.6. Statistical analysis

First, we evaluated how trends in population-normalized ZEV adoption, from 2015–2023, varied by gentrification and DAC status in the San Francisco Bay and Southern California metropolitan areas. We fit a linear mixed-effects model for $ZE{V}_{ij}$, population-normalized ZEV for census tract *i* in year *j*, which included census tract-level random intercepts (${b}_{0i}$), and fixed effects for a binary indicator of DAC status at 2015 (${d}_{i}$; reference: non-DAC), indicators of gentrification status (${g}_{i}^{\left(1\right)},\,{g}_{i}^{\left(2\right)}$; reference: ‘none/early’), and a smooth function of time, $s\left({t}_{ij}\right)$, using a natural cubic spline of year with 7 degrees of freedom, selected to minimize Bayesian Information Criteria (BIC). To allow the nonlinear time trends to vary by gentrification and DAC, we also included two- and three-way interactions. The model was:

\begin{eqnarray*}\begin{array}{lll}ZE{V}_{ij} &amp; = &amp; \left({\beta }_{0}+{b}_{0i}\right)+{\beta }_{d}{d}_{i}+{\beta }_{g}^{\left(1\right)}{g}_{i}^{\left(1\right)}+{\beta }_{g}^{\left(2\right)}{g}_{i}^{\left(2\right)}+s\left({t}_{ij}\right)\\ &amp; &amp; \,+{\beta }_{dg}^{\left(1\right)}{d}_{i}{g}_{i}^{\left(1\right)}+{\beta }_{dg}^{\left(2\right)}{d}_{i}{g}_{i}^{\left(2\right)}+{d}_{i}{s}_{d}\left({t}_{ij}\right)+{g}_{i}^{\left(1\right)}{s}_{g}^{\left(1\right)}\left({t}_{ij}\right)+{g}_{i}^{\left(2\right)}{s}_{g}^{\left(2\right)}\left({t}_{ij}\right)\\ &amp; &amp; \,+{d}_{i}{g}_{i}^{\left(1\right)}{s}_{dg}^{\left(1\right)}\left({t}_{ij}\right)+{d}_{i}{g}_{i}^{\left(2\right)}{s}_{dg}^{\left(2\right)}\left({t}_{ij}\right)+{\varepsilon }_{ij}\end{array}\end{eqnarray*}

Second, we related changes in ZEV adoption to subsequent changes in housing prices, in the subset of census tracts classified as none/early-gentrifying (n = 1,975; 29.65%). Changes in ZEV adoption were calculated, at the census tract-level, as the difference from 2015 to 2019 in population-normalized BEV (and PHEV) and then categorized by quartiles, with cut points of 0.296, 0.792, and 1.82 for BEV (and 0.546, 1.18, 2.14 for PHEV). Figure S.4 presents the spatial distribution of those changes in ZEV adoptions.

We visualized trends in housing prices using LOESS, separately for median home value and monthly rent, across quartiles of change in BEV (and PHEV). We fit linear mixed effects models to relate prior change in ZEV (${\mathrm{\Delta }}ZE{V}_{i}$ from 2015–2019) to subsequent time-varying housing prices (${y}_{ij}$) from 2020 to 2023, using a linear trend for time as follows:

\begin{eqnarray*}\begin{array}{lll}{\mathrm{log}}\,{y}_{ij} &amp; = &amp; \left({\beta }_{0}+{\beta }_{1}{\mathrm{\Delta }}ZE{V}_{i}^{\left(Q2\right)}+{\beta }_{2}{\mathrm{\Delta }}ZE{V}_{i}^{\left(Q3\right)}+{\beta }_{3}{\mathrm{\Delta }}ZE{V}_{i}^{\left(Q4\right)}+{b}_{0i}\right)\\ &amp; &amp; \,+\left({\beta }_{4}+{\beta }_{5}{\mathrm{\Delta }}ZE{V}_{i}^{\left(Q2\right)}+{\beta }_{6}{\mathrm{\Delta }}ZE{V}_{i}^{\left(Q3\right)}+{\beta }_{7}{\mathrm{\Delta }}ZE{V}_{i}^{\left(Q4\right)}+{b}_{1i}\right){t}_{ij}+\ldots +{\varepsilon }_{ij}\end{array}\end{eqnarray*}

where housing prices, ${y}_{ij}$, were natural log transformed prior to modeling due to left-skew; ${t}_{ij}$ is year centered at 2020; ${\mathrm{\Delta }}ZE{V}_{i}^{\left(Q2\right)}$, ${\mathrm{\Delta }}ZE{V}_{i}^{\left(Q3\right)}$, and ${\mathrm{\Delta }}ZE{V}_{i}^{\left(Q4\right)}$ are indicators for quartiles of prior change in ZEV, with Q_1_ as the reference; and census tract-level random effects are $\left({b}_{0i},\,{b}_{1i}\right)\,\sim \,{\mathrm{M}}VN\left(0,\,{\mathrm{\Psi }}\right)$. By including a time fixed effect ${t}_{ij}$, we also adjusted for time trends, such as inflation and overall price growth. Figure S.5 presents the spatial distribution of changes in median home value and rent from 2020 to 2023, providing geospatial context for housing market trends. To understand the sensitivity of models to covariate adjustments, we fit a series of models with sequential covariate adjustment (denoted as ‘…’ above), including: an unadjusted model; models adjusting separately for time-varying population size, median income, or education (percent of adults over age 25 with a bachelor’s degree); and an income and education adjusted model.

For interpretability, model results were translated back into average annual USD change in housing prices per ${\mathrm{\Delta }}ZEV$ quartile. Details expanded in Supplementary Method.

All analyses were conducted in R version 4.5.0 [31].

## 3. Results

Results are presented for two main analyses. Sections 3.1–3.3 describe trends in ZEV adoption across neighborhoods at different stages of gentrification and DAC status. The second analysis relates prior changes in ZEV adoption to subsequent changes in housing prices in None/early gentrified census tracts (sections 3.4–3.5). Results are reported separately by ZEV type.

### 3.1. Gentrification status and DAC

Among the study sample (6,447 census tracts), 1,975 (30.63%) were classified as None/Early, 2,030 (31.49%) as Advanced/Stable, and 2,442 (37.88%) as Exclusive. Among None/Early tracts, 54.68% (n = 1,080) were designated as DACs, compared to 17.44% (n = 354) in Advanced/Stable and 6.72% (n = 164) in Exclusive tracts (table 1). DACs in Exclusive tracts often experienced higher pollution levels.

**Table 1.** Cross-tabulation of Gentrification status and disadvantaged communities (DAC) among California census tracts with gentrification status based on typologies defined by Urban Displacement Project (2000–2018).

	None/Early N (col %)	Advanced/Stable N (col %)	Exclusive N (col %)	Total N (row %)
Non-DAC	888 (44.96%)	1675 (82.51%)	2273 (93.08%)	4836 (75.01%)
DAC	1080 (54.68%)	354 (17.44%)	164 (6.72%)	1598 (24.79%)
Missing CalEnviroScreen scores	7 (0.35%)	1 (0.05%)	5 (0.20%)	13 (0.20%)
Total (row %)	1975 (30.63%)	2030 (31.49%)	2442 (37.88%)	6447

### 3.2. ZEV adoption trends

Overall, ZEV adoption increased from 2015 to 2023, with BEVs experiencing a higher and sharper rise than PHEVs (figure S.3). For example, in 2015, median BEV per 1000 population among study neighborhoods was 0.91 (interquartile range, IQR: 0.25–2.55), and increased to 14.97 (IQR: 6.14–30.93) in 2023. In contrast, median PHEV per 1000 population increased from 1.03 (IQR: 0.32–2.59) in 2015 to 4.61 (IQR: 2.39–7.74) in 2023. BEV adoption accelerated after 2020, while PHEV growth plateaued (figure S.6). A slight slowdown in BEV adoption was observed between 2021 and 2022.

### 3.3. Trends in ZEV adoption by gentrification status and DAC

**Figure 1.**
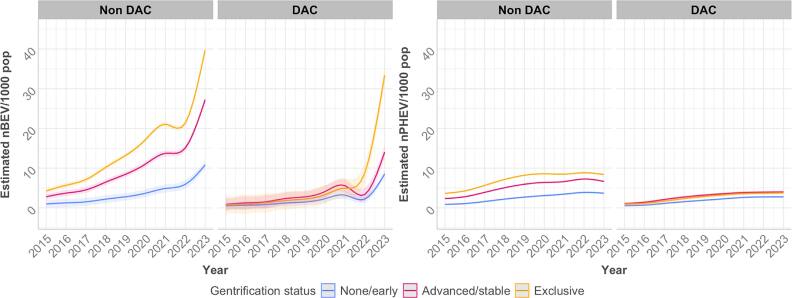
Census tract BEV and PHEV adoption from 2015 to 2023 in California, USA, by disadvantaged community (DAC) status and baseline gentrification status, with trends (solid lines) and 95% Confidence Intervals (shaded areas) estimated using a linear mixed-effects model.

The predicted ZEV per 1,000 population was lower among DACs compared to non-DACs for both BEV and PHEV (figure 1). Among non-DACs, BEV and PHEV adoption trends differed by gentrification status across the whole study period, with Exclusive neighborhoods having a higher and sharper increase (e.g., nBEV per 1000 population from 4.24 [95% CI: 3.58–4.91] in 2015 to 39.72 [39.05–40.39] in 2023), followed by Advanced/Stable (2.79 [2.02–3.58] to 27.23 [26.45,28.01]) and None/early neighborhoods (0.83 [−0.86–2.53] to 14.04 [0.87–12.34]). Among DACs, differences were smaller with overlapping confidence intervals and crossed trends between Advanced/Stable and Exclusive neighborhoods (figure 1). None/early gentrified neighborhoods remain low in ZEV adoption in both DAC and non-DAC.

### 3.4. Trends in housing prices and ZEV adoption among none/early gentrified neighborhoods

**Figure 2.**
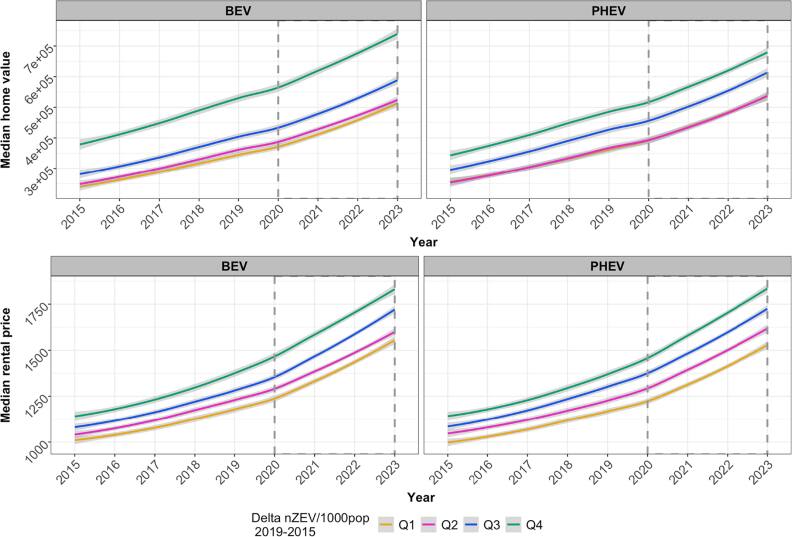
Trends in median home values and monthly rental prices (USD) from 2015 to 2023 in None/Early gentrified neighborhoods by quartiles of change in ZEVs per 1,000 population (2015–2019), stratified by ZEV type. Gray boxes focus on the study period (2020–2023). Lines represent loess-smoothed trends; shaded areas indicate 95% confidence intervals. Note that in the PHEV-home value plot, overlap of Q1 and Q2 makes only Q2 visible.

A total of 1,975 (29.65%) census tracts were assigned as None/early. In these tracts, median home values rose steadily from $288,545 in 2015 to $575,000 in 2023, while median monthly rents increased from $1,056 to $1,653. Trends in housing prices from 2020 to 2023 followed an approximately linear pattern across quartiles of prior 5-year change in population-normalized ZEVs (figure 2). Tracts in the highest quartile (Q_4_) had consistently higher median housing prices, followed by Q_3_, Q_2_, and Q_1_, although trends of home values between Q_2_ and Q_1_ overlapped for PHEV (figure 2).

### 3.5. Association between ZEV adoption and housing prices

Spatially, census tracts with the largest increases in ZEV adoption were clustered in affluent areas, including the SF Bay shoreline and west side of LA, whereas the smallest increases were concentrated in economically marginalized areas such as the eastern Bay Area and southeast Los Angeles (figure S.4). Median home values and rents increased most strongly in shoreline and core urban areas of the Bay Area as well as in the City of LA (figure S.5).

#### 3.5.1. BEV adoption and home values

**Figure 3.**
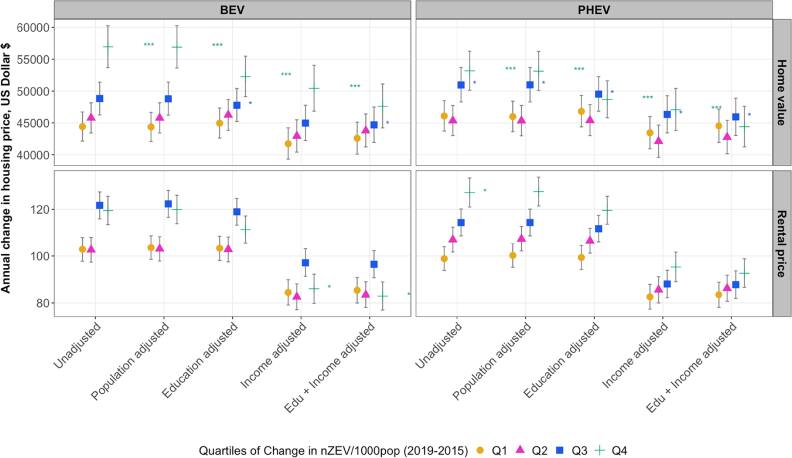
Annual average change (USD) in median home values and monthly rental prices from 2020 to 2023 in None/Early gentrified neighborhoods for quartiles of change in nZEVs per 1,000 population (2015–2019) by ZEV type. Estimates are derived from linear mixed-effects models with the following specifications: (1) unadjusted (no other covariates); (2) adjusted for population size (population adjusted); (3) adjusted for educational attainment (% with a college degree); (4) adjusted for median household income (income adjusted); and (5) education and income adjusted (edu+income adjusted). Asterisks indicate significance of the interaction term between time and quartiles of change in nZEV/1000pop (*p < 0.05, **p < 0.01, **p < 0.001).

Census tracts in the highest quartile of prior 5-year change in BEV (Q_4_) had statistically significantly greater annual average change in median home values compared to the lowest quartile (Q_1_) ($47,615 [44,219–51,108] versus $42,584 [40,097–45,127] in the fully adjusted model) (Table A.1). On a relative scale, however, Q_4_ experienced an 8.69% [8.12%–9.28%] increase compared to 10.8% [10.3%–11.4%] in Q_1_ (Table S.1). Changes among Q_3_ and Q_2_ showed a stepwise pattern, and the associations were attenuated after adjusting for education and income (figure 3).

#### 3.5.2. BEV adoption and rental prices

Census tracts in the highest quartile of prior 5-year change in BEV (Q_4_) had a statistically significant lower annual average monthly rental price increase compared to Q_1_ ($82.9 [76.9, 88.9] versus $85.4, [80.0–90.8]), but only in the income and education adjusted model (table A.1). In the model adjusted for income only, Q_4_ had a slightly higher increase compared to Q_1_; however, note that both estimates had wide, overlapping confidence intervals (figure 3). Census tracts in Q_3_ consistently showed higher increases in rental prices than those in Q_1_, although the differences were not statistically significant across models (figure 3).

#### 3.5.3. PHEV adoption and home values

PHEV adoption in Q_4_ was associated with a statistically significant higher annual average change in home values compared to Q_1_ ($47,071 [43,813–50,421] versus $43,454 [40,930–46,011] in the model adjusted for income). Differences were similar when the model was adjusted for both education and income ($44,398 [41,237–47,608] versus $44,537 [41,933-47,162]) (figure 3, table A.1). Q_3_ tracts also displayed a significantly higher annual average increase than Q_1_, with estimates largely overlapping with those of Q_4_ (figure 3). Similar to BEV, those increases on the absolute scale were a decrease on the relative scale (table A.1).

#### 3.5.4. PHEV adoption and rental prices

For median monthly rental price, a stepwise pattern was observed, with Q_4_ associated with the highest increase and Q_1_ the lowest (figure 3). However, the finding was only statistically significant in the unadjusted model. These results were consistent across absolute and relative scales (table A.1).

## 4. Discussion

This real-world longitudinal study was motivated by concerns raised by community partners and the EVAC advisory council. Results supported that DAC experienced inequity in ZEV adoption, and even among non-DAC there are large differences in ZEV adoption by gentrification status. Disadvantaged and non- or early-gentrifying neighborhoods lag in ZEV adoption in California metropolitan areas. Among None/early gentrifying tracts, higher prior BEV and PHEV adoption was associated with larger subsequent increases in home values. Although this study does not establish causality, our findings reinforce the community concerns regarding how ZEV-related investments may intersect with ongoing gentrification and displacement pressures. A key aspect of our second analysis is its focus on None/early neighborhoods. Many of these areas have historically been underinvested in and have incomes below the state average. In this context, increased ZEV adoption does not necessarily coincide with neighborhood change, making these neighborhoods an important setting for examining these patterns. Moreover, the limited prior real-world evidence on this topic, our study underscored the value of applying a community-informed model to help identify and address underexplored but in-need research questions.

### 4.1. Equitable adoption and gentrification

California leads the nation in ZEV adoption [32] and has pioneered ZEV mandates and sales requirements to promote uptake [33, 34]. Yet we find substantial adoption gaps between DACs and non-DACs, consistent with prior evidence of slower ZEV uptake in disadvantaged neighborhoods [1, 6], and severe income inequality in PHEV ownership [35]. While a qualitative study identified cost and charging access as key factors influencing ZEV adoption decisions in those communities [12], studies reported that non-White and low SES households were less likely to access rebates and incentives [36, 37], and faced limited EV infrastructure [35, 38, 39], compounding existing barriers. Recent national survey evidence suggests that income does not significantly moderate the relationship between factors such as commuting behavior, parking and charging access, or vehicle ownership and EV purchase intent, though baseline disparities in access to parking and charging infrastructure persist for low-income households [40]. More broadly, differences in ZEV adoption may also depend on regional energy systems. In California, electricity generation has shifted toward a cleaner mix over time [41]. However, access to and benefits from renewable and low-carbon sources may vary systematically across disadvantaged communities.

ZEV adoption also varied by gentrification stage. Census tracts categorized as Advanced/Stable or Exclusive at baseline adopted ZEVs faster than None/Early tracts These gentrification statuses are closely linked to SES [29, 42, 43] and likely capture socioeconomic advantages that facilitate adoption. More gentrified neighborhoods may also benefit from more developed infrastructure, supporting quicker uptake [38, 39]. This observation aligns with Diffusion of Innovations theory [44], where acceptance of ZEV technology starts slowly by motivated and resourceful early adopters, then scales up when cost and barriers are lowered.

We also observed distinct BEV and PHEV adoption trends. Before 2018, adoption trends were similar, with PHEVs appealing to early EV users through extended range and flexibility [45, 46]. However, over time, interest shifted toward BEVs, potentially reflecting higher incentives [47], improved leasing options [48], improved ranges, and growing satisfaction among previous PHEV users [49]. The a slowdown from 2021 to 2022 reflects broader market trends during that period [50].

### 4.2. Association with home values in none/early gentrified neighborhoods

In neighborhoods not undergoing gentrification or only in early stages, higher ZEV adoption (especially in top quartile) was associated with greater absolute increases in median home values. Although this specific association has not been documented before, several plausible pathways have been identified. First, EV infrastructure, such as EVCS, can contribute to increases in local property values [3, 51], and tends to cluster within isolated land-use zones favoring privileged communities [52]. In California, public EVCS installation was associated with a 3.3% increase in house sales price premiums within a 1 km radius [21]. These value gains may also contribute to neighborhood change. Study cautioned that charging infrastructure may accelerate gentrification by influencing housing markets [51]. Public EV investments can stimulate policy and living interests in neighborhoods, raising housing prices [53]. Rising home costs have been linked with loss of social capital, including social cohesion and networks, that may consequently displace disadvantaged residents [54]. Second, EV-related transportation investments often require parking and may be more compatible with lower-density housing [3, 55]. These spatial and infrastructural demands can further increase property values.

Higher ZEV adoptions may also reflect ongoing gentrification that itself raising housing prices. Neighborhoods at risk of gentrification usually have more vacant properties and are near higher-income, more affluent areas with fewer vacancies [56–58], making them attractive to investments and higher-income in-migrants who are likely ZEV owners. Their arrival raises both ZEV ownership and housing prices, ultimately displacing long-term, lower-income residents [59].

The scale of the comparison, absolute or relative, matters. When interpreting our results on a relative scale, the percentage increase in home values was actually smaller in higher-adopting areas than in the lowest quartile. This is because higher-adopting neighborhoods had higher baseline values, possibly reflecting higher socioeconomic position, which constrained relative growth. We prioritized reporting absolute increases as our main findings, as this better accounts for these baseline differences and reflects overall change. This distinction between absolute and relative change should be considered when interpreting results.

### 4.3. Association with rental prices in none/early gentrified neighborhoods

Rental prices could be affected by EV adoption through pathways similar to those for home values. However, unlike home values, rental prices did not have consistent or meaningful differences across quartiles of ZEV adoption during the study period. Several factors may explain this pattern. First, multi-family housing, commonly used for rentals, may be less directly affected by EV-related infrastructure investments. These properties often face greater barriers to EV charger installation due to affordability concerns, parking configurations, limited electrical capacity, and renovation constraints [60, 61]. Second, statewide rental regulations may play a role. California’s Tenant Protection Act of 2019 (AB 1482), which took effect on January 1, 2020, caps annual rent increases at 5% plus local inflation or a maximum of 10%, whichever is lower [62]. This policy may have limited rental price growth even in areas with higher ZEV adoption.

### 4.4. Chicken-or-egg question

It is important to note that our study was not designed to establish causal relationships, i.e., the ‘chicken-or-egg’ question: does ZEV adoption drive gentrification, or does gentrification facilitate higher apparent ZEV adoption? Our study demonstrates associations, not causal directions. Several constraints limit our ability to draw such conclusions.

First, gentrification is a complex, multi-stage process that unfolds over extended periods [29, 42]. Most studies assess it over 10 years [43], whereas we examined 5-year changes in ZEV adoption and 4-year changes in housing prices, which may not capture the full temporal extent of neighborhood transformation. Additionally, changes in both ZEV and housing prices might reflect underlying gentrification, i.e., greater ZEV adoption itself may be part of gentrification and then contribute to rising housing prices. Given this complexity, our study design could not distinguish between the causes and effects of gentrification.

Second, although our dataset captures temporal changes in ZEV adoption and housing prices, it lacks direct measures of population or demographic shifts (e.g., whether higher-income ZEV owners moving into the neighborhood). It also remains unclear whether rising housing prices reflects an influx of higher-income households, improved conditions for existing residents, or survey responses changes [63]. When controlling for gentrification-related variables (e.g., income, education), associations attenuated, suggesting ZEV adoption may reflect broader SES shifts, including gentrification itself. Prior studies show that income growth in low-income areas often results from in-migration of higher-income households [64]. Future research may use income shifts as a measure for such in-migration.

Finally, while we used home value and rental price, common components of gentrification indices [65], these proxies cannot fully represent the gentrification dynamics. How gentrification was conceptualized also affects findings [29, 66]. For instance, a California study showed that emphasizing housing affordability in gentrification metrics revealed harmful associations with severe maternal morbidity, whereas population-based definitions indicated protective effects [27]. We selected housing-related variables because transportation investments are known to influence housing prices [15, 21, 67]. We aimed to use these indicators for observational purposes and acknowledge that they do not fully capture the broader social and demographic processes of gentrification.

### 4.5. Strengths and limitations

Our study has several strengths. First, it advances the literature on equitable ZEV adoption by examining its relationship with gentrification and housing prices, which has thus far received little attention. While higher ZEV adoption in economically advantaged areas may appear intuitive, cross-sectional analyses cannot capture how adoption patterns intersect with neighborhood changes. By focusing on gentrification status and changes in housing prices, this study highlights ZEV adoption and urban/housing dynamics that are not observable from static SES measures alone. Our findings open new topics for future research and raise important policy considerations as California continues to expand ZEV adoptions. Second, we utilized publicly available, real-world longitudinal data to assess associations over time. This design helps mitigate time-invariant confounding within census tracts, enhancing validity. Third, since our study period crossed the census boundary change, we harmonized ACS and ZEV data using a crosswalk [28] to a consistent geographic scale, reducing data loss and minimizing bias due to changing tract boundaries [68, 69]. Last, our study was motivated by a research question raised directly by community partners, highlighting the importance of community engagement in transportation equity research [16].

This study has limitations. First, we relied on a single gentrification measure (UDP), selected because it is publicly available, ready for use in California, and captures housing market changes relevant to our research question. Alternative socioeconomic-based approaches [29, 66] were not considered. Notably, a San Francisco-based study compared three indicators and found that UDP effectively identified changing urban tracts and provided early markers of gentrification, including areas at risk [70], the very area of interest in our study. Second, categorizing changes in ZEV adoption into quartiles, while useful for interpretability and for exploring potential non-linear associations, resulted in a loss of information. Some quartiles, such as Q_2_ for BEV, have a narrow range (0.296 to 0.792 per 1,000 population), which may limit its discriminatory power. Future studies might explore alternative parametrizations for better quantifying the differences. Third, we relied on ACS 5-year estimates for housing price measurements. Because annual values are based on overlapping 5-year samples, they introduce temporal correlation [71], potentially underestimating uncertainty and narrowing confidence intervals. To address this, we included census tract-level random intercepts and year-specific random slopes as a standard approach for correlated data. While this approach partially accounts for correlation, reduced year-to-year variability in ACS data may overstate precision. Despite its value for tracking neighborhood trends, ACS data have known accuracy limitations [63]. Future studies should consider more temporally precise housing data. Fourth, renter-dominated, not gentrified/in early stages neighborhoods may have home value estimations based on small samples, increasing margins of error. Lastly, our study focused on California metropolitan areas with available UDP typologies. Caution is warranted in generalizing these findings to non-metropolitan regions or other states, because gentrification dynamics, housing price inflation trajectories, and ZEV adoption patterns vary substantially by context. As such, the results should be interpreted in the context of the study regions’ housing trends.

### 4.6. Research and policy implications

Our findings raise equity concerns and have implications for policies aimed at accelerating transportation electrification. If increases in ZEV adoption are linked to rising housing costs, efforts to accelerate the electrification of transportation could unintentionally exacerbate existing disparities. However, ZEV investments are not easily linked to housing stability or affordability, especially since much of the charging infrastructure is residential. Ensuring that communities most vulnerable to pollution and environmental health burdens benefit from the ZEV transition will require complementary policies beyond transportation policy alone. In areas with housing market pressure, policymakers may need to consider coordinating with housing and land-use agencies to avoid unintended consequences. Scholars proposed strategies to address these challenges, such as expanding affordable housing, engaging community stakeholders, and equalizing access to high-quality transportation [16].

At the same time, while this study does not establish causality, determining causal relationships will be critical to address the concerns raised by community partners. Future research should prioritize causal inference and investigate the underlying pathways linking ZEV adoption and neighborhood change. Doing so will require access to data at finer spatial resolutions and over longer timeframes [43, 72], as well as interdisciplinary collaborations to tackle this complex question [43, 73]. In addition to quantitative work, qualitative research is needed to understand how residents perceive and experience the interaction between transportation electrification and displacement pressures.

## Data Availability

The data that support the findings of this study are openly available at the following URL. See manuscript citation for full URL/DOIs. The analysis code that supports the findings of this study is openly available at: https://github.com/madaopt/EVxGen-a-longitudinal-study-in-California.

## References

[1] Garcia E, Johnston J, McConnell R, Palinkas L, Eckel S P (2023). California’s early transition to electric vehicles: observed health and air quality co-benefits. Sci. Total Environ..

[2] Gujral H, Franklin M, Easterbrook S (2025). Emerging evidence for the impact of Electric Vehicle sales on childhood asthma: can ZEV mandates help?. Environ. Res..

[3] Henderson J (2020). EVs are not the answer: a mobility justice critique of electric vehicle transitions. Annals of the American Association of Geographers.

[4] Pennington A F, Cornwell C R, Sircar K D, Mirabelli M C (2024). Electric vehicles and health: a scoping review. Environ. Res..

[5] Hennessy E M, Syal S M (2023). Assessing justice in California’s transition to electric vehicles. IScience.

[6] Yu Q, He B Y, Ma J, Zhu Y (2023). California’s zero-emission vehicle adoption brings air quality benefits yet equity gaps persist. Nat. Commun..

[7] OEHHA (2022). https://oehha.ca.gov/calenviroscreen/sb535.

[8] Loustaunau M G, Chakraborty J (2019). Vehicular air pollution in Houston, texas: an intra-categorical analysis of environmental injustice. International Journal of Environmental Research and Public Health.

[9] Miranda M L, Edwards S E, Keating M H, Paul C J (2011). Making the environmental justice grade: the relative burden of air pollution exposure in the United States. International Journal of Environmental Research and Public Health.

[10] Griswold S K, Nordstrom C R, Clark S, Gaeta T J, Price M L, Camargo J C A (2005). Asthma exacerbations in North American adults: who are the ‘frequent fliers’ in the emergency department?. Chest.

[11] Jones R, Lin S, Munsie J P, Radigan M, Hwang S-A (2008). Racial/ethnic differences in asthma-related emergency department visits and hospitalizations among children with wheeze in Buffalo, New York. Journal of Asthma.

[12] Palinkas L A, Liff M, Campos A, Martinez A A, Eckel S P, Chen F, Johnston J, Franco W, Garcia E (2025). A co-created, community-informed model for electric vehicle adoption in disadvantaged communities. Transportation Research Part D: Transport and Environment.

[13] Cohen M, Pettit K L (2019). https://www.urban.org/research/publication/guide-measuring-neighborhood-change-understand-and-prevent-displacement.

[14] Dawkins C, Moeckel R (2016). Transit-induced gentrification: Who will stay, and who will go?. Housing Policy Debate.

[15] Fleming K L (2018). Social equity considerations in the new age of transportation: electric, automated, and shared mobility. Journal of Science Policy & Governance.

[16] Freemark Y, Rennert L, McDaniel N, Su Y, Finio N, Harvey C, Knaap G, Dawkins C, Somashekhar S, Cabral L (2025). https://urban.org/sites/default/files/2025-07/Gentrification%20Guide%20-%20NCHRP08-160IR_0.pdf.

[17] Balboni C, Bryan G, Morten M, Siddiqi B (2020). https://web.stanford.edu/~memorten/ewExternalFiles/CCMS_TZ.pdf.

[18] Bouzarovski S, Frankowski J, Tirado Herrero S (2018). Low-carbon gentrification: When climate change encounters residential displacement. International Journal of Urban and Regional Research.

[19] Delmelle E C (2021). Advances in Transport Policy and Planning.

[20] Rice J L, Cohen D A, Long J, Jurjevich J R (2020). Contradictions of the climate-friendly city: new perspectives on eco-gentrification and housing justice. International Journal of Urban and Regional Research.

[21] Liang J, Qiu Y, Liu P, He P, Mauzerall D L (2023). Effects of expanding electric vehicle charging stations in California on the housing market. Nature Sustainability.

[22] CARB (2025). https://arb.ca.gov/emfac/fleet-db.

[23] California Energy Comission (2025). https://energy.ca.gov/data-reports/energy-almanac/zero-emission-vehicle-and-infrastructure-statistics-collection/new-zev.

[24] OEHHA (2023). https://oehha.ca.gov/calenviroscreen/report/calenviroscreen-40.

[25] Chapple K, Thomas T, Zuk M (2021). Urban Displacement Project Website.

[26] Thomas T, Hartmann C, Driscoll A, Chapple K, Cash A, Elias R R, Zuk M (2020). https://urbandisplacement.org/wp-content/uploads/2021/07/udp_replication_project_methodology_10.16.2020-converted.pdf.

[27] Gao X, Thomas T A, Morello-Frosch R, Allen A M, Snowden J M, Carmichael S L, Mujahid M S (2023). Neighborhood gentrification, displacement, and severe maternal morbidity in California. Social Science & Medicine.

[28] Manson S, Schroeder J, Riper D V, Knowles K, Kugler T, Roberts F, Ruggles S (2024). IPUMS National Historical Geographic Information System: Version 19.0 [dataset].

[29] Finio N (2022). Measurement and definition of gentrification in urban studies and planning. Journal of Planning Literature.

[30] Wilhelmsson M, Ismail M, Warsame A (2022). Gentrification effects on housing prices in neighbouring areas. International Journal of Housing Markets and Analysis.

[31] R Core Team (2025). R Foundation for Statistical Computing.

[32] California Energy Comission (2025). https://energy.ca.gov/news/2025-01/californias-zev-momentum-rolls-2025.

[33] Long Z, Axsen J, Kitt S (2020). Public support for supply-focused transport policies: Vehicle emissions, low-carbon fuels, and ZEV sales standards in Canada and California. Transportation Research Part A: Policy and Practice.

[34] Governor of California (2020). https://gov.ca.gov/wp-content/uploads/2020/09/9.23.20-EO-N-79-20-Climate.pdf.

[35] Lee D-Y, Wilson A, McDermott M H, Sovacool B K, Kaufmann R, Isaac R, Cleveland C, Smith M, Brown M, Ward J (2025). Does electric mobility display racial or income disparities? Quantifying inequality in the distribution of electric vehicle adoption and charging infrastructure in the United States. Appl. Energy.

[36] Rubin D, St-Louis E (2016). Evaluating the economic and social implications of participation in clean vehicle rebate programs: who’s in, who’s out?. Transp. Res. Rec..

[37] Ku A L, Graham J D (2022). Is California’s electric vehicle rebate regressive? a distributional analysis. Journal of Benefit-Cost Analysis.

[38] Hsu C-W, Fingerman K (2021). Public electric vehicle charger access disparities across race and income in California. Transport Policy.

[39] Lou J, Shen X, Niemeier D A, Hultman N (2024). Income and racial disparity in household publicly available electric vehicle infrastructure accessibility. Nat. Commun..

[40] Kim J D, Bozeman I I I J, F, Carley S, Konisky D M, Matisoff D C, Michalek J J, Nock D (2025). Beyond the cost: Electric vehicle ownership and adoption intent in US households. Transportation Research Part D: Transport and Environment.

[41] California Energy Comission (2025). https://energy.ca.gov/data-reports/energy-almanac/california-electricity-data/electric-generation-capacity-and-energy.

[42] Shaw K (2008). Gentrification: what it is, why it is, and what can be done about it. Geography Compass.

[43] Bhavsar N A, Kumar M, Richman L (2020). Defining gentrification for epidemiologic research: a systematic review. PLoS One.

[44] Rogers E M, Singhal A, Quinlan M M (2014). An Integrated Approach to Communication Theory and Research.

[45] Lane B W, Dumortier J, Carley S, Siddiki S, Clark-Sutton K, Graham J D (2018). All plug-in electric vehicles are not the same: predictors of preference for a plug-in hybrid versus a battery-electric vehicle. Transportation Research Part D: Transport and Environment.

[46] Liao F, Molin E, Van Wee B (2017). Consumer preferences for electric vehicles: a literature review. Transport Reviews.

[47] Roberson L, Helveston J P (2022). Not all subsidies are equal: measuring preferences for electric vehicle financial incentives. Environ. Res. Lett..

[48] Liao F, Molin E, Timmermans H, van Wee B (2019). Consumer preferences for business models in electric vehicle adoption. Transport Policy.

[49] Lee J H, Cho M, Tal G, Hardman S (2023). Do plug-in hybrid adopters switch to battery electric vehicles (and vice versa)?. Transportation Research Part D: Transport and Environment.

[50] Lazo A (2024). https://calmatters.org/environment/2024/02/california-electric-cars-industry-slowdown/.

[51] Russo S, Spiller B, Wilwerding R (2024). Equity in Electric Vehicle Charging Infrastructure.

[52] Carlton G J, Sultana S (2022). Electric vehicle charging station accessibility and land use clustering: a case study of the Chicago region. Journal of Urban Mobility.

[53] Zuk M, Bierbaum A H, Chapple K, Gorska K, Loukaitou-Sideris A (2018). Gentrification, displacement, and the role of public investment. Journal of Planning Literature.

[54] Lees L (2008). Gentrification and social mixing: towards an inclusive urban renaissance?. Urban studies.

[55] Namdeo A, Tiwary A, Dziurla R (2014). Spatial planning of public charging points using multi-dimensional analysis of early adopters of electric vehicles for a city region. Technol. Forecast. Soc. Change.

[56] Maantay J A, Maroko A R (2018). Brownfields to greenfields: Environmental justice versus environmental gentrification. International Journal of Environmental Research and Public Health.

[57] Richardson J, Mitchell B, Franco J (2019). Shifting neighborhoods: gentrification and cultural displacement in American cities.

[58] Lee R J, Newman G (2021). The relationship between vacant properties and neighborhood gentrification. Land Use Policy.

[59] Guerrieri V, Hartley D, Hurst E (2013). Endogenous gentrification and housing price dynamics. Journal of Public Economics.

[60] Pierce L, Bui A (2024). https://theicct.org/wp-content/uploads/2024/04/ID-23-%E2%80%93-MFH-charging-Working-Paper-letter_final.pdf.

[61] Kandhra D, MacCurdy D, Lipman T (2024). https://escholarship.org/content/qt9dn2j441/qt9dn2j441.pdf.

[62] California Legislative Information (2019). https://leginfo.legislature.ca.gov/faces/billTextClient.xhtml?bill_id=201920200AB1482.

[63] Immergluck D, Hollis A (2024). Different data, different measures: comparing alternative indicators of changes in neighborhood home values. Housing Policy Debate.

[64] Comandon A, Rodnyansky S, G. Boarnet M (2024). Who drives neighborhood income growth? an analysis of new versus long-term residents in the Northern California Megaregion. Journal of Planning Education and Research.

[65] Preis B, Janakiraman A, Bob A, Steil J (2021). Mapping gentrification and displacement pressure: an exploration of four distinct methodologies. Urban Studies.

[66] Smith G S, Breakstone H, Dean L T, Thorpe J R J (2020). Impacts of gentrification on health in the US: a systematic review of the literature. Journal of Urban Health.

[67] Yiu C Y, Wong S K (2005). The effects of expected transport improvements on housing prices. Urban Studies.

[68] Thomas M (2021). https://blogs.library.duke.edu/data/2021/03/25/standardizing-the-u-s-census/.

[69] Schroeder J P (2007). Target-density weighting interpolation and uncertainty evaluation for temporal analysis of census data. Geogr. Anal..

[70] Mujahid M S, Sohn E K, Izenberg J, Gao X, Tulier M E, Lee M M, Yen I H (2019). Gentrification and displacement in the San Francisco Bay area: a comparison of measurement approaches. International Journal of Environmental Research and Public Health.

[71] Herman E (2008). The American community survey: an introduction to the basics. Gov. Inf. Q..

[72] Easton S, Lees L, Hubbard P, Tate N (2020). Measuring and mapping displacement: The problem of quantification in the battle against gentrification. Urban Studies.

[73] Cole H V (2020). A call to engage: considering the role of gentrification in public health research. Cities & Health.

